# Emergent biotechnology applications in urology: a mini review

**DOI:** 10.3389/fbioe.2025.1539126

**Published:** 2025-02-04

**Authors:** Chang Liu, Alejandro Rivera Ruiz, Yingchun Zhang, Philippe Zimmern, Zhengwei Li

**Affiliations:** ^1^ Department of Biomedical Engineering, University of Houston, Houston, TX, United States; ^2^ Department of Biomedical Engineering, University of Miami, Coral Gables, FL, United States; ^3^ Department of Urology, The University of Texas Southwestern, Dallas, TX, United States; ^4^ Department of Biomedical Sciences, The Tilman J. Fertitta Family College of Medicine, University of Houston, Houston, TX, United States

**Keywords:** living cells, whole-cell biosensors, optogenetics, bioengineered urinary bladder, 3D bioprinting, urological conditions

## Abstract

Technological advances have significantly impacted the field of urology, providing innovative solutions for diagnosis, treatment, and management of various urological disorders and diseases. This article highlights four groundbreaking technologies: whole-cell biosensors, optogenetic interventions for neuromodulation, bioengineered urinary bladder, and 3D bioprinting. Each technology plays a crucial role in enhancing patient care and improving clinical outcomes in urology. Advances in these fields underscore a shift towards precision diagnostics, personalized treatments, and enhanced regenerative strategies, ultimately aiming to enhance patient outcomes and address unmet clinical needs in urological diseases.

## 1 Introduction

**FIGURE 1:**
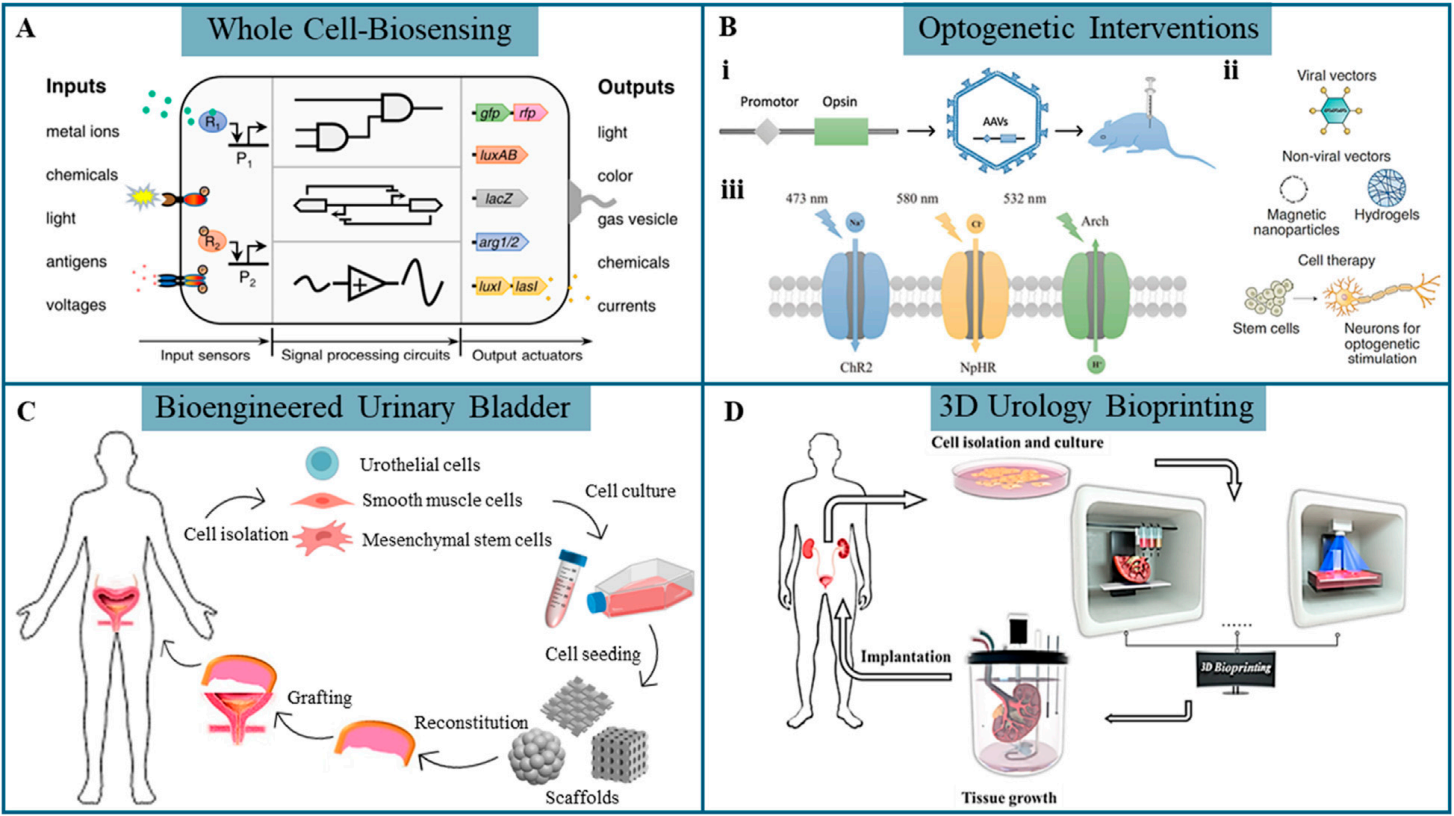
Emergent biotechnologies in urology. **(A)** Synthetic cell technology is used to develop whole-cell biosensors capable of detecting specific biological, stimuli (Wan et al., 2021). **(B)** (i) Viral vector transduction for inducing specific opsin expression, opsins (iii) (Zhou and Liao, 2021) and targeted gene delivery (ii) in optogenetics (Bansal et al., 2023). **(C)** Strategies for bioengineered urinary bladder (Orabi et al., 2013). **(D)** 3D bioprinting approaches for urological reconstruction (Xu et al., 2022).

The urinary system, comprising the kidneys, ureters, bladder, and urethra, is essential for maintaining homeostasis through the removal of metabolic waste, regulation of blood pressure and volume, electrolyte balance, and acid-base equilibrium (Flores et al., 2023). These functions are critical for physiological stability and overall health. However, dysfunctions and diseases of the urological system, including chronic kidney disease, bladder dysfunction, and urinary tract infections, can result in serious complications such as renal failure, incontinence, and increased susceptibility to systemic infections (Dirks et al., 2006). The prevalence and impact of these conditions highlight the urgent need for advanced methods of early detection, diagnosis, and treatment. Living cells are pivotal in biomedical engineering, offering unique capabilities that facilitate the development of precision therapies and diagnostic toolkits, promising early detection and effective treatment (Bianco and Robey, 2001; Moraskie et al., 2021; Bansal et al., 2023; Zhang Y. S. et al., 2024). Cells play a transformative role in advanced applications such as whole-cell biosensors (WCBs), optogenetic interventions for neuromodulation, bioengineered urinary bladder, and 3D urology bioprinting, providing novel solutions for managing urological health, dysfunction, and diseases (Figure 1).

WCBs utilize living cells as the primary sensing elements for detecting various biological or chemical substances (e.g., metabolites (Liu et al., 2015) and metal ions (Guo et al., 2018; Zhang J. et al., 2024)) as well as physical signals (e.g., electrical signals (Bhokisham et al., 2020), temperature (Inda et al., 2019) and pressure (Fajardo-Cavazos et al., 2012)). Engineered microbial or mammalian cells respond to specific analytes by expressing detectable reporter genes such as colorimetric, fluorescent, or bioluminescent markers, enabling real-time monitoring (Figure 1A) (Wan et al., 2021). A typical WCB comprises a genetic reporter linked to a sensing unit, which regulates the expression of a detectable output, and can be integrated with multiple sensors. In comparison to traditional biosensors, WCBs offer superior sensitivity and cost-effectiveness due to the inherent molecular recognition capabilities of the cells. Several WCBs have been developed to detect biomarkers in blood and urine, aiding in the diagnosis and risk assessment of kidney-related disorders (O'Connor et al., 2016; Lubkowicz et al., 2018; Reyes et al., 2020).

Optogenetics, a powerful and precise technique, involves genetically engineering cells to express light-sensitive ion channels or proteins, allowing precise control of cellular behavior through specific light wavelengths (Zhou and Liao, 2021). Optogenetic techniques are fundamentally defined by three key components: the gene encoding light-sensitive proteins (such as opsins) to be transferred, the target tissue or cells for genetic modification, and the gene delivery vector, which facilitates the introduction of the gene into the target cells. Viral transduction is the common gene targeting method in optogenetic interventions, particularly utilizing adeno-associated viruses (AAVs) and lentiviruses (Figure 1Bi) (Zhou and Liao, 2021). Alternatively, non-viral vectors, including nanoparticles and hydrogels, offer viable options for gene delivery, though their application is less established (Figure 1Bii) (Bansal et al., 2023). Transgenesis, another gene-targeting strategy, involves complex methodologies and is not suitable for human applications due to significant technological and ethical challenges. By targeting cells modified with opsins using controlled light pulses, optogenetics enables high-precision neuromodulation. Channelrhodopsin 2 (ChR2), the most widely used depolarizing opsin activated by blue light at 473 nm, triggers ion influx and neuron activation, while hyperpolarizing opsins, such as Natronomonas pharaonis halorhodopsins (NpHR) and Archaerhodopsin (Arch), inhibit neuronal activity by inducing a more negative cellular charge upon light activation (Figure 1Biii) (Zhou and Liao, 2021). Co-expressing multiple opsins with distinct light sensitivities in the same cell enables precise and selective control over cellular activation and inhibition (Keshmiri Neghab et al., 2022). The use of optogenetics to address bladder dysfunctions, such as overactive bladder (DeBerry et al., 2018), underactive bladder (Zhou et al., 2024), and Detrusor Sphincter Dyssynergia (Hong et al., 2023), has garnered significant interest and holds promise for targeted neuromodulation.

Bioengineered urinary bladder remains a key focus area in regenerative engineering, with strategies aiming to create functional equivalents of urinary tissues for augmentation cystoplasty and bladder defect repair (Wang et al., 2022). Figure 1C illustrates strategies for tissue-engineered urinary bladder regeneration (Orabi et al., 2013). Cellular grafts combine biomaterials with living cells to cultivate neo-tissues *in vitro*. Cells such as urothelial cells (UCs), smooth muscle cells (SMCs), or stem cells are seeded onto scaffolds that mimic the extracellular matrix (ECM), providing essential mechanical and biochemical cues to support cell growth and differentiation. The engineered tissue is subsequently implanted to promote *in vivo* tissue repair and regeneration. Although substantial advancements have been achieved, vascularization and innervation of biomaterials remain critical challenges. Existing engineered tissues are predominantly inert and lack the necessary functionality for complete integration and regeneration.

3D bioprinting, a cutting-edge technique, enables the layer-by-layer construction of tissue-like structures using living cells embedded in bioinks. Cells are printed in biocompatible hydrogels, providing structural support and an optimal growth environment. Common 3D bioprinting techniques include inkjet-based, extrusion-based, and laser-assisted bioprinting, such as digital light processing and stereolithography. 3D bioprinting holds significant promise for creating complex, vascularized tissue constructs, including urological organs like the kidney, bladder, and urethra (Figure 1D) (Xu et al., 2022). As an emerging technology, 3D bioprinting opens potential applications in personalized medicine, drug screening, or even whole-organ replacement.

Living cells are at the forefront of advancements in urology, enabling innovations that mimic, modify, or potentially replace natural biological functions to enhance human health and therapeutic capabilities. The following sections present a selection of representative studies from emerging research across four key areas, highlighting the functionality and versatility of living cells in urological applications and offering a comprehensive overview of the diverse strategies being developed to address challenges in the field of urology.

## 2 Whole-cell biosensors for urological diagnostics

**FIGURE 2:**
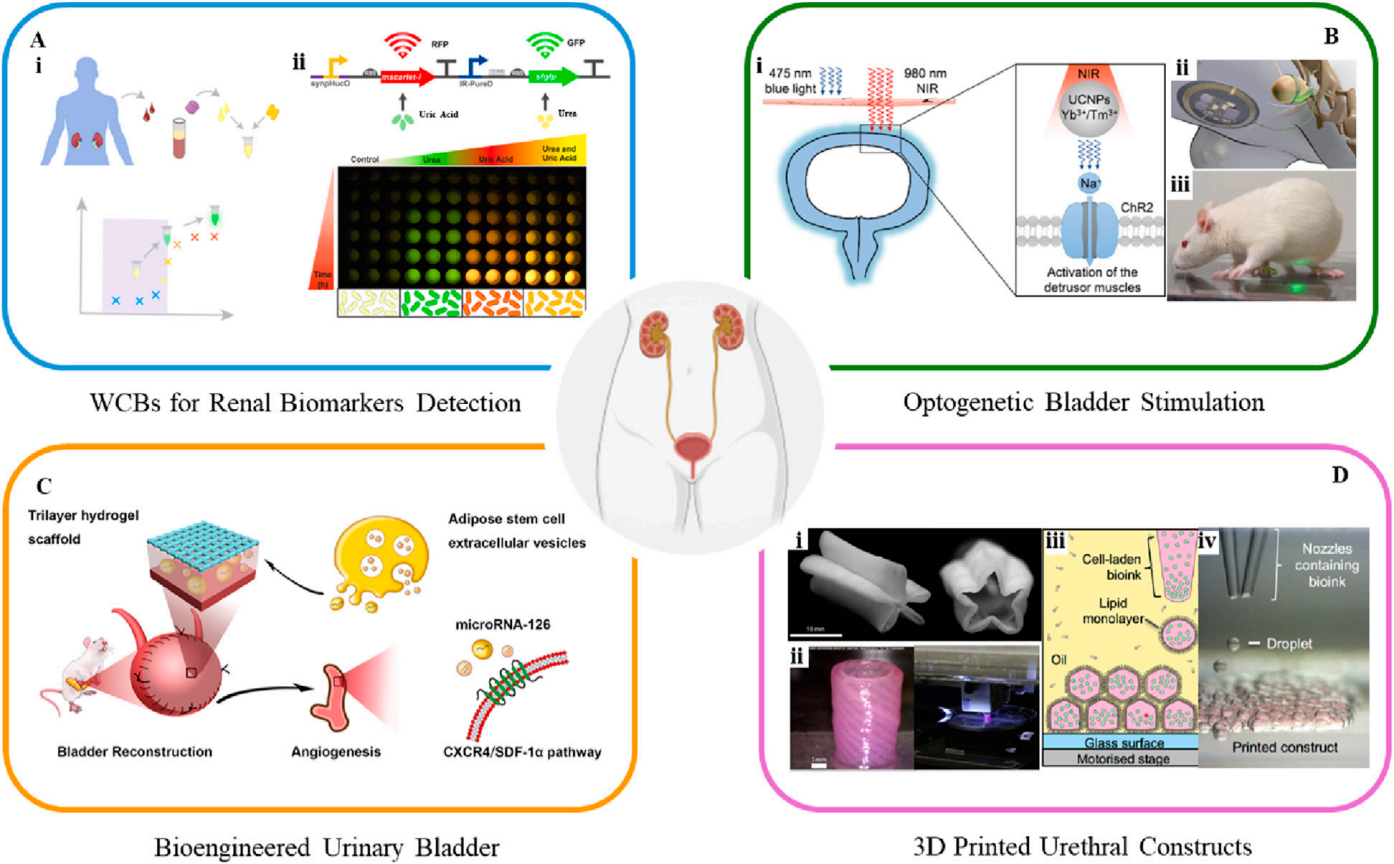
A class of cutting-edge platforms for urological applications. **(A)** Workflow of whole-cell biosensors for detecting renal biomarkers (Kose et al., 2023). **(B)** Schematic of a (i) UCNP-mediated optogenetic system (Zhou et al., 2024) and (ii, iii) advanced wireless light-delivery devices for bladder control (Mickle et al., 2019). **(C)** Schematic of human ASCs-EVs-encapsulated BAMG-HS scaffold delivering miR-126 to promote angiogenesis in bladder regeneration via CXCR4/SDF-1α activation (Xiao et al., 2021). **(D)** 3D bioprinted (i) star-shaped scaffold in closed and partially open position, mimicking urination (Versteegden et al., 2017), and (ii) PCL/PLCL urethral constructs (Zhang et al., 2017). (iii, iv) 3D bioprinting pattern human embryonic kidney cells and ovine mesenchymal stem cells in high droplet resolution of 1 nL (Graham et al., 2017).

Several biomarkers found in blood and urine serve as indicators to diagnose urological diseases. Urine, in particular, contains biomarkers that reflect a wide range of health conditions. In response to the growing demand for early and accessible detection of kidney health, recent advancements have focused on the development of portable, cost-effective biosensing platforms. Researchers have engineered a bacterial WCB with an optoelectronic measurement module to detect heme, a component of lysed red blood cells found in urine, which serves as an early biomarker for kidney disease (Barger et al., 2021). This biosensor uses *Escherichia coli* modified with a heme-sensitive synthetic promoter linked to the *luxCDABE* luciferase reporter gene from *Photorhabdus luminescens*. Enhanced sensitivity is achieved by splitting the *luxCDABE* operon, allowing *luxCDE* expression to be regulated by the heme-sensitive promoter, while *luxAB* is controlled by either a constitutive or inducible promoter. When tested in human urine with lysed blood, this biosensor, combined with a single-photon avalanche photodiode-based detection system, shows promise as a low-cost, portable diagnostic tool. Another electrochemical WCB using *Bacillus licheniformis* measures urea concentration in urine samples, reaching a sensitivity of 1.278 μA/M with a detection limit of 0.01 M, offering a reliable method for monitoring urea levels critical for kidney function assessment (Ariyanti et al., 2020). Researchers have also developed biosensors for detecting urea and uric acid, incorporating advanced gene expression control strategies to improve sensitivity and response profiles (Kose et al., 2023). These systems integrate an AND-logic gate for dual biomarker detection, enabling multiplexed measurements in complex biological human serum samples (Figure 2Ai). The dual reporter system, where urea induces sfGFP expression and uric acid induces mScarlet-I expression, demonstrates distinct, time-dependent fluorescence responses for both analytes (Figure 2Aii). The biosensor proves effective, confirming its potential for low-cost, personalized healthcare applications, although further optimization is needed for clinical implementation.

Overall, bacterial WCBs represent a reliable platform for detecting target substances (e.g., heme, urea, and uric acid), offering advantages over conventional methods and gaining attention for potential applications in kidney function assessment and disease monitoring. While challenges related to biocontainment, specificity, and prolonged response times persist, WCBs show considerable promise for advancing precision diagnostics and individualized treatment strategies.

## 3 Optogenetic interventions for neuromodulation

Optogenetics is an emerging non-invasive technology that uses light-sensitive proteins to precisely control and monitor cell behavior, showing significant potential in regulating bladder storage and voiding for urinary bladder control. Optogenetic stimulation of urothelial cells can directly induce bladder contractions, as demonstrated in a study using a uroplakin II (UPK2) cre mouse model combined with a ChR2 expressing mouse (Robilotto et al., 2023). Activation of UCs in UPK2-ChR2 mice leads to cell depolarization, ATP release, and increased bladder pressure and pelvic nerve activity, observed through cystometry recordings. This approach demonstrates optogenetics' potential in inducing bladder contractions and advancing the understanding of urothelial-to-sensory neuron communication and pathophysiology. A recent study explores optogenetics for bladder modulation, using ChR2 to induce contractions in bladder SMCs via 475 nm blue light (Zhou et al., 2024). To overcome tissue penetration limitations, this study further develops a minimally invasive method using upconversion nanoparticles (UCNPs) with 980 nm near-infrared (NIR) light, where the UCNPs emit light at 475 nm when excited at 980 nm to activate ChR2 (Figure 2Bi). The approach activates ChR2 successfully, inducing cation influx and successfully inducing bladder contractions noninvasively. *Ex vivo* and *in vivo* tests show improved detrusor pressure and micturition volume with NIR-induced voiding. This UCNPs-mediated optogenetics method offers high spatial specificity, making it a promising treatment in the management of a neurogenic underactive bladder. Moreover, it is worth noting that a miniaturized bio-optoelectronic implant utilizing an optical stimulation interface that leverages microscale inorganic light-emitting diodes to activate opsins has been developed (Figures 2Bii, iii) (Mickle et al., 2019). The integration of bioelectronics with optogenetic techniques has led to wireless, closed-loop systems for monitoring and treating bladder function, thereby expanding the scope of optogenetics in urology.

Optogenetics exhibits potential to resolve deficiencies in traditional treatment such as sacral neuromodulation, by specifically activating or inhibiting the target cells to eliminate off-target effects or reveal underlying causes of ineffective neuromodulation. Optogenetics also holds great value in elucidating the structures and functions of neural circuits involved in bladder control. However, challenges remain, including the need for improved light delivery systems to address tissue penetration and biocompatibility concerns, as well as ensuring the long-term stability and safety of optogenetic tools in clinical applications.

## 4 Bioengineered urinary bladder

Bioengineered urinary bladder aims to replace or restore damaged tissue using regenerative medicine approaches. Current strategies involve a combination of stem cell therapy and biomaterial scaffolds which support the growth and integration of bladder tissue (Serrano-Aroca et al., 2018). Living cells are increasingly employed in bladder regeneration, often in combination with advanced biomaterials. Autologous cells are preferred for seeding to minimize inflammatory responses and prevent rejection. UCs and SMCs can be harvested through biopsy, expanded *in vitro*, and re-implanted into the same host (Sharma and Basu, 2022). However, using autologous bladder cells for graft preparation is not always feasible, as it may require additional surgery, increase morbidity, and lead to potential issues such as abnormal cell growth and poor cell adhesion, especially in patients with underlying pathological conditions. In cases where suitable host tissue biopsies are unavailable, stem cells are being explored as promising alternatives for regeneration (Adamowicz et al., 2017; Xiao et al., 2017; An et al., 2020). The selection of scaffold types is also critical, as the cell type and scaffold should complement each other. For example, a tri-layer scaffold combines a bladder acellular matrix graft with alginate dialdehyde and gelatin hydrogel, reinforced by a degummed silk mesh (BAMG-HS), effectively addressing challenges of collagen scaffolds, such as suboptimal clinical outcomes and inadequate smooth muscle regeneration (Xiao et al., 2021). Encapsulation of adipose-derived stem cells (ASCs) within the scaffold demonstrates cytocompatibility and mechanical properties, supporting bladder tissue regeneration and functional recovery. Additionally, human ASCs-derived extracellular vesicles (EVs) activate the CXCR4/SDF-1α pathway, leading to VEGF secretion and facilitating both morphological and functional recovery following bladder augmentation (Figure 2C).

Despite these advances, challenges remain in translating these techniques into clinical practice, particularly regarding the biocompatibility, mechanical durability, and bio-scaffold design and optimization to ensure adequate tissue vascularization and innervation for long-term functionality. As tissue-engineered constructs advance toward clinical use, 3D bioprinting technologies offer new possibilities for fabricating complex, functional bladder models and organoid systems.

## 5 3D bioprinting in urology

3D bioprinting has emerged as a transformative tool for creating patient-specific constructs for urological reconstructive surgery and organ replacement (Xu et al., 2022). In addition to biomaterial composition and cell type, the architecture and topology of tissue engineering scaffolds play a critical role in tissue regeneration. 3D bioprinting overcomes geometry limitations by enabling the creation of complex, layered architectures with location-specific biomechanical properties. The technology provides high resolution, structural complexity, and material heterogeneity, making it ideal for replicating the diverse organ structures and ECM compositions of urological tissues, such as the kidney, bladder and urethra, thereby having the potential to facilitate personalized tissue regeneration and precise cell delivery (Booth et al., 2024). Figure 2Di illustrates a collagen-based, star-shaped scaffold seeded with UCs and cultured under dynamic conditions simulating urination, created using direct extrusion bioprinting (Versteegden et al., 2017). This scaffold exhibits increased burst pressure and mechanical integrity after 1000 cycles, with 75% UC coverage, emphasizing the importance of hydrodynamic factors in mimicking native urethral behavior. Figure 2Dii presents a tubular scaffold made from poly(ε-caprolactone) (PCL)/poly(lactide-co-caprolactone) (PLCL), designed to replicate the structural and mechanical properties of urethral tissue via a layer-by-layer deposition technique (Zhang et al., 2017). The integration of cell-laden fibrin hydrogel is proposed to enhance the microenvironment, promoting cell growth. The bioprinting system successfully produces a tubular scaffold, with UCs and SMCs evenly distributed in the inner and outer layers, respectively. Although the *in vitro* formation of the cell-laden constructs is successful, they have not been tested in an animal model. Figures 2Diii, iv show human embryonic kidney (HEK) cells and ovine mesenchymal stem cells (oMSCs) printed using inkjet-based bioprinting with agarose-based bioink at tissue-relevant densities (10^7^ cells/ml) and a droplet resolution of 1 nL (Graham et al., 2017). High-resolution 3D geometries are successfully fabricated, with printed cells demonstrating high viability, HEK cell proliferation, and oMSCs differentiating into cartilage-like structures expressing type II collagen after 5 weeks. The proliferation of HEK cells within the printed structures suggests the potential for developing tissue-like constructs for kidney regeneration.

Overall, 3D bioprinting technology has been employed to create cell-laden urologic constructs by integrating various polymer types with scaffold designs and structural characteristics. This approach has proven effective in replicating the structure and mechanical properties of some urinary organs. Nevertheless, challenges remain, including ensuring the long-term stability and functionality of printed constructs, achieving sufficient vascularization and innervation, and establishing reliable *in vivo* models.

## 6 Conclusion and future perspectives

Living cells exhibit versatile properties in urological applications, offering significant promise while also presenting persistent challenges. Bacterial whole-cell biosensors (WCBs) have been developed to detect analytes with high sensitivity, offering a cost-effective and noninvasive diagnostic tool with controlled treatment potential. However, design challenges, such as issues with biocontainment, specificity, and extended response times remain. Despite these limitations, advancements in synthetic biology toolkits are driving the evolution of living sensor platforms, positioning them as promising analytical devices capable of meeting real-world detection needs with superior sensitivity and reduced cost. Continued fundamental research is essential to identify new biomarkers and sensor elements, which would support the development of standardized genetic building blocks for biosensors.

Optogenetics, a promising modality for bladder neuromodulation, holds potential for treating bladder pathologies. Key considerations for optogenetic applications include selecting appropriate opsins, gene-targeting methods, and light-delivery strategies. Fortunately, the integration of bioelectronics with optogenetic techniques, exemplified by bio-optoelectronic implants employing optical stimulation interfaces, leverages micro–light-emitting diodes (µ-LEDs) to activate opsins (Mickle et al., 2019; Jang et al., 2020). Future advancements in bio-optoelectronic interfaces, wireless platforms, and nanoparticle-based light delivery could further refine optogenetics, making it a transformative technology for managing neurogenic bladder disorders.

Bioengineered urinary bladder, particularly those utilizing stem cells, represent a promising future for reconstructive urology, with high potential for clinical translation. Multifunctional biomaterial-assisted scaffolds are central to bioengineered bladder by providing the mechanical and biochemical cues necessary for cell growth and differentiation. Hydrogels, with their 3D network structure and hydrophilic composition, closely resemble the natural ECM and are particularly suited to support cellular environments. Stem cell-based bioengineered bladder also minimizes foreign body responses through immunomodulatory properties such as mesenchymal stem cells (MSCs), which actively interact with the immune system to reduce inflammation and promote tissue integration around implanted biomaterials.

Advances in 3D bioprinting further enhance the possibilities for generating complex, high-resolution, and heterogeneous structures, making this technology ideal for creating the organ structures and ECM compositions found in urologic structures such as the kidney, bladder, and urethra, thus contributing to personalized tissue regeneration and precise cell delivery. Although 3D bioprinting holds immense promise for urological applications, it faces significant challenges, particularly regarding the scalability of the printing process. Developing larger and more complex constructs demands precise cell alignment and adequate vascularization to support functional tissue regeneration. Future research is expected to emphasize advancing bioprinting technologies to improve tissue maturation, refine cell-material interactions, and establish robust *in vivo* models, ultimately validating the therapeutic potential of bioprinted urological constructs for clinical translation.

In conclusion, genetically engineered cells are central to advancing WCBs and optogenetics, establishing them as key components in urological applications. The development of cell-seeded (particularly human induced pluripotent stem cell (iPSC)-based) bioengineered urinary bladders and 3D bioprinting of urological scaffolds offers substantial benefits, including reduced immunogenicity and the potential for personalized treatment. While each technology presents unique opportunities to enhance patient outcomes, challenges in implementation and optimization remain. Ongoing interdisciplinary collaborations between engineers, biologists, and clinicians will be crucial to realizing the full potential of these innovations, ultimately enhancing patient care and outcomes in urology.
